# Survivin Measurement improves Clinical Prediction of Transition From Arthralgia to RA—Biomarkers to Improve Clinical Sensitivity of Transition From Arthralgia to RA

**DOI:** 10.3389/fmed.2018.00219

**Published:** 2018-08-02

**Authors:** Malin C. Erlandsson, Minna Turkkila, Rille Pullerits, Maria I. Bokarewa

**Affiliations:** ^1^Department of Rheumatology and Inflammation Research, Institute of Medicine, University of Gothenburg, Gothenburg, Sweden; ^2^Rheumatology Clinic, Sahlgrenska University Hospital, Gothenburg, Sweden; ^3^Department of Clinical Immunology and Transfusion Medicine, Sahlgrenska University Hospital, Gothenburg, Sweden

**Keywords:** clinical trials, outcome measures, rheumatoid arthritis, arthralgia, biomarkers, survivin

## Abstract

**Background:** Arthralgia often predates development of rheumatoid arthritis (RA). A set of joint symptoms commonly found in patients during their transition from arthralgia to RA, has been recently proposed.

**Aim:** To combine clinical and serological markers and to improve recognition of imminent rheumatoid arthritis (RA) among patients with arthralgia.

**Methods:** The total of 1,743 first-visit patients attending the rheumatology ward in Gothenburg for joint symptoms were identified during 12 consecutive months. Among those, 63 patients were classified as RA, 73 had undifferentiated arthritis and 180 had unexplained arthralgia. New RA cases, which prospectively developed during 48 months, comprised the preclinical (pre) RA group. The joint symptoms of the first-visit were analyzed aiming to distinguish patients with arthralgia and arthritis, and patients with pre-RA, who later developed the disease. The receiver operating characteristics curves were constructed. In the model, symptoms with the odds ratio >2.0 between the arthralgia and pre-RA were combined with information about RA-specific antibodies, C-reactive protein (CRP), and survivin in serum.

**Results:** The proposed set of clinical symptoms distinguished the arthralgia patients from RA and pre-RA. Presence of survivin in serum showed strong association with clinical joint symptoms in arthralgia. A combination of symptoms in several small joint areas, increasing number of joints with symptoms, and patient's experience of swelling in small hand joints at the first visit identified pre-RA cases with 93% specificity. Grouping those symptoms with information about survivin, RA-specific antibodies, and CRP (or gender) in the final algorithm achieved 91% specificity and 55.2% of positive prediction for transition from arthralgia to RA.

**Conclusion:** Clinical and serological parameters in combination aid recognition of imminent RA among arthralgia patients with appropriate sensitivity.

## Introduction

Patients with musculoskeletal complains comprise the major part of visitors in general medical practice. Observational studies in Europe and the USA report a delayed referral of RA patients by general practitioners (1–3). Patients with undiagnosed RA visited their general practitioners an average of four times before being referred to rheumatologist (4). Studies of the recent years convincingly showed that early recruitment of RA patients from general clinical practice and initiation of anti-rheumatic treatment improved health outcomes, saved the quality adjusted life years (5), and slowed down irreversible consequences of arthritis (6–9).

Development of musculoskeletal symptoms, as arthralgia or tendinitis, often precedes clinical arthritis. Indeed, a progress to RA occurred predominantly among individuals with musculoskeletal symptoms (10–12), while prospective monitoring of asymptomatic relatives of RA patients revealed a low absolute frequency of new RA cases (13–16). Thus, specific characteristic of musculoskeletal complains may give an opportunity to clinically identify the individuals at risk for progression to RA. The EULAR working group has recently summarized the expertise gained by rheumatologists and presented a set of parameters defining clinically suspect arthralgia (17). As indicated by the definition, a combination of those parameters would aid recognition of individuals at preclinical phase of RA. The set of parameters was developed by the modified Delphi consensus approach where the clinical expertise was the reference. Finally, a longitudinal validation study undertaken by the members of the EULAR taskforce showed only a limited positive predictive value of the clinical parameters, when used as designed, and stressed requirement of biomarkers for high specificity on imminent RA (18).

Measurement of RA-specific antibodies allowed to limit the vast group of patients with musculoskeletal complains. It has been established that RA-specific antibodies may be detected years before the disease onset and when combined with musculoskeletal symptoms, present patients at risk for development of RA (19, 20). Most of the cohort studies selected patients by presence of rheumatoid factor (RF) and/or antibodies to cyclic citrullinated peptides (ACPA) and confirmed that presence of these antibodies was associated with the development of arthritis (10, 21, 22). The results of these studies made obvious that even this careful pre-selection of risk cohort had low prevalence of incidental RA. Additionally, new cases of RA occurred among patients with no measurable antibodies almost with the same frequency.

Other serological markers have been evaluated in the preclinical phase of RA and include acute phase reactants C-reactive protein and erythrocyte sedimentation rate, cytokines and chemokines. The obtained results are conflicting; some studies identified their association with arthritis development (23–26), others have not (11, 27, 28).

In this study we focus on predictive potential of serum detection of oncoprotein survivin. Survivin has originally been described in hematological malignancies and in solid tumors, and since than it serves as an established marker of malignant cell transformation (29). It appeared to be a multifunctional protein with numerous roles in non-malignant processes (30). Being a part of chromosomal passenger complex, it participates in cell division supporting renewal of non-malignant tissues (31–33). By inhibiting apoptosis and regulating bioprocessing of micro-RNA, survivin has important role in cell differentiation. It protects the pool of pluripotent hematopoietic and neuronal progenitors (34–36). In adaptive immunity, it regulates development of antigen presenting cells, functional T cell populations and supports formation of immunological memory (37–41).

In RA, survivin is expressed in the synovial macrophages, T cells of the inflamed joints and B cells of the bone marrow. It promotes transition of synovial fibroblasts to an invasive phenotype followed by proliferation of synovial tissue and pannus formation (42–44). Not surprising, high levels of survivin in serum are associated with severe course of joint disease, development of skeletal damage and resistance to anti-rheumatic treatment (45–47). It has also been associated with major known risk factors for the development of RA including carriage of HLA-DRB1 antigen (48), smoking (49, 50) and production of RA-specific antibodies (23, 46, 48). Similar to RA-specific antibodies, survivin may be measured in preclinical phase of RA (51). Inhibition of survivin alleviates development of experimental arthritis and significantly reduces joint damage (52).

In a recent population-based study within two university cities in Sweden, we demonstrated that measurement of survivin individually or in combination with RA-specific antibodies improves estimation of RA risk and prospectively predicts RA development in patients with arthralgia (53). Taking advantage of this arthralgia cohort, we designed a prospective study aiming to validate utility of the consensus-based set of clinical characteristics of arthralgia tending to progress to RA and to match symptoms with serological biomarkers for imminent RA.

## Materials and methods

### Participants and study design

The inception cohort consisted of the patients referred for the first assessment to the Rheumatology Clinic at the Sahlgrenska University Hospital of Gothenburg by general practitioners between November 5, 2012-November 4, 2013. The medical records of the first visit were carefully sorted in two steps by experienced rheumatologists (RP, MB). During the first step, all patients with known diagnosis of inflammatory joint or spine disease, systemic rheumatic disease, gout and generalized pain conditions were excluded. During the second step, the patients with arthritis were separated from those with musculoskeletal complains that could not be explained by an obvious joint swelling. The latter patients comprised the group of arthralgia (Table 1). Inclusion into the study required the medical record of the first visit to a rheumatologist and the presence of a blood sample in adherence to that visit. We excluded the individuals below 18 years of age and those who have been registered deceased for the time point of their medical records evaluation.

**Table 1:** Characteristics of the study cohorts.

**Characteristics**	**Arthralgia *n* = 180**	**UA *n* = 73**	**RA *n* = 63**
Women, *n* (%)	145 (80.6)	51 (69)	44 (69.8)
Age, years, Median [IQR]	48 [36-58]	53 [33-65]	55 [47-67] *^*P* < 0.001^*
Current smoker, *n* (%)	45 (25)	16 (22)	25 (40) *^*P* = 0.031^*
EULAR/ACR2010 score, median [IQR]	2 [1-3]	3 [3-4]	7 [6-8]
Increased CRP, *n* (%)	54 (30)	Not assessed	28/55 (33)
Survivin-positive, *n* (%)	61 (34)	20 (27)	31 (49)
Any autoantibody positive, *n* (%)	53 (29)	10 (14)	50 (79)
RF positive, *n* (%)	39 (16)	10 (14)	36 (57)*^*P* = 0.034^*
ACPA positive, *n* (%)	12 (7)	1 (1)	30 (48)*^*P* < 0.001^*

*P-values indicate differences between RA and arthralgia groups. UA, undifferentiated arthritis; RA, rheumatoid arthritis; IQR, Inter-quartile range; EULAR/ACR, epidemiologic criteria for RA approved by the European League against Rheumatism and the American College of Rheumatology at 2010; CRP, C-reactive protein; ACPA, antibodies to citrullinated peptides; RF, rheumatoid factor*.

The study was reviewed and approved by the Ethical Review Board of Gothenburg. The trial is registered at www.ClinicalTrials.gov and received the identification number NCT03444623.

### Clinical data collection

Medical records of the first and all consecutive visits for the period of 48 months were reviewed by two independent assessors (SB and SK) blinded to the serological data and to clinical out-come of the patients. The review was structured after the set of parameters describing clinically suspect arthralgia proposed by the EULAR expert taskforce at the Delphi phase II (17). The following clinical symptoms were included in the list: (1) joint symptoms onset <1 year, (2) 4–10 joints with symptoms, (3) symptoms located in MCPs, (4) symptoms located in MTPs, (5) symptoms located in several small joint regions, (6) symmetric symptoms and signs, (7) morning stiffness ≥60 min, (8) most severe symptoms in early morning, (9) improvement of symptoms during the day, (10) increasing number of joints with symptoms over time, (11) patients experience of swollen small hand joints, (12) presence of a first degree relative with RA, (13) local tenderness involved joints at physical examination.

The assessors were instructed and trained to keep a keen eye for clinical signs of swollen joints as identified by rheumatologists. If signs of joint swelling were present, the EULAR/ACR 2010 Epidemiological RA Criteria form (54) was completed for the first clinical visit and at the follow-up visits. Information regarding the presence and location of tender and swollen joints, acute phase reactants erythrocyte sedimentation rate (ESR) and C-reactive protein (CRP), duration of symptoms and results of RF and ACPA was summarized. The patients with joint swelling and fulfilling 6 points of the EULAR/ACR2010 criteria comprised the RA group, while the remaining patients were classified as undifferentiated arthritis (UA). All the patients with arthralgia were followed longitudinally to arthritis within the period of 48 months. The patients with arthralgia who on the prospective follow-up developed joint swelling and fulfilled the RA criteria were considered pre-RA at their first visit. The family history of arthritis and smoking habits were retrieved from medical records based on patient self-reports.

### Serological analyses

Detection of anti-cyclic citrullinated peptide antibodies (ACPA) was performed with automatic multiplex method for anti-CCP2 antibodies (BioPlex® 2,200 System, BioRad, Hercules, CA, USA). The levels of ACPA in each sample were quantified against the calibration curve. The cut-off level above 20 AU/ml was regarded positive. This corresponded to the mean+2SD of the healthy control group. Total RF, antibodies against Fc-region of gamma globulin, was measured by nephelometry (Beckman Immage 800, Beckman Coulter AB, Brea, CA, USA). The levels of total RF were presented in U/ml. The cut-off for RF positivity was 20 U/ml. The measurements of ACPA and RF were performed at the accredited laboratory of Clinical Immunology, Sahlgrenska University Hospital.

Serum levels of CRP were measured at the Laboratory of Clinical Chemistry, Sahlgrenska University Hospital, by nephelomety (Beckman Immage 800). The cut-off level for positivity was 5 mg/L.

Serum levels of survivin were measured in samples diluted 1:10 using a sandwich enzyme-linked immunoassay comprising a pair of matched antibodies and recombinant standard (DY647, R&D Systems, Minneapolis, MN USA); the detection limit was 100 pg/ml. The cut-off level for survivin positivity was set to 450 pg/ml corresponding to the mean+3SD of 104 healthy individuals (39).

### Clinical outcomes and statistical analysis

The primary clinical outcome of this study was development of polyarthritis meeting 6 points and more of the EULAR/ACR Criteria 2010 (55).

The data were retrieved from clinical records aiming to complete the set with clinical parameters suggested by the EULAR taskforce (17). Information about squeeze test of MCP and MTP joints and difficulty at making a fist was missing in at least 50% of the cases. Those parameters were excluded from further analysis. Dataset of the remaining clinical symptoms comprised 1.98% of missing data. Multiple imputation was performed in SPSS to complete these missing data. To explore which of these clinical parameters predicted future RA in arthralgia patients, a chi-square analysis was done for each parameter. The clinical parameters with the odds ratio >2.0 between the arthralgia and pre-RA groups were included into a model and receiver operating characteristics (ROC) curves were constructed. The area under the curve (AUC) was analyzed and completed with information about sensitivity and specificity. To improve the sensitivity of the model for RA, the parameters of potential relevance such as present smoking, female gender, age above 50, high serum levels of CRP, survivin and autoantibodies were one by one included into the model.

Statistical calculations were performed using the SPSS, version 21 (IBM SPSS, Chicago, IL), GraphPad prism version 6, and www.openepi.com/Menu/OE_Menu.htm for two-by-two tables. Data is presented as median [IQR] or in absolute numbers. Continuous data were compared by non-parametric analyses using Mann-Whitney U test when appropriate. A two-tailed P value of < 0.05 was considered statistically significant.

## Results

### Clinical characteristics of joint symptoms in patients with arthralgia and arthritis

Among the medical records of the 1743 first-visit patients, we identified 136 cases of new arthritis. Among those, 63 fulfilled the EULAR/ACR 2010 classification criteria for RA and the remaining 73 cases were classified as UA. The expected incidence of new RA cases is 0.1–0.5/103 inhabitants (54). This inception cohort was retrieved from 513 751 inhabitants of Gothenburg, giving the estimated incidence of new RA between 51–257 cases/year. Thus, the annual incidence of RA and UA cases identified by the survey corresponded to the expected in Gothenburg. Additional 180 individuals with joint complains but no obvious swollen joints at the first visit comprised the group with unexplained arthralgia. Demographic characteristics of the patient cohort are presented in Table 1.

The analysis of joint symptoms at the first visit revealed that the overall frequency of these symptoms was lowest among the arthralgia patients, followed by UA and RA. This numerical increase in clinical symptoms clearly separated the arthralgia patients from the patients with inflammatory arthritis, RA and UA (both, *P* < 0.001).

**Figure 1:**
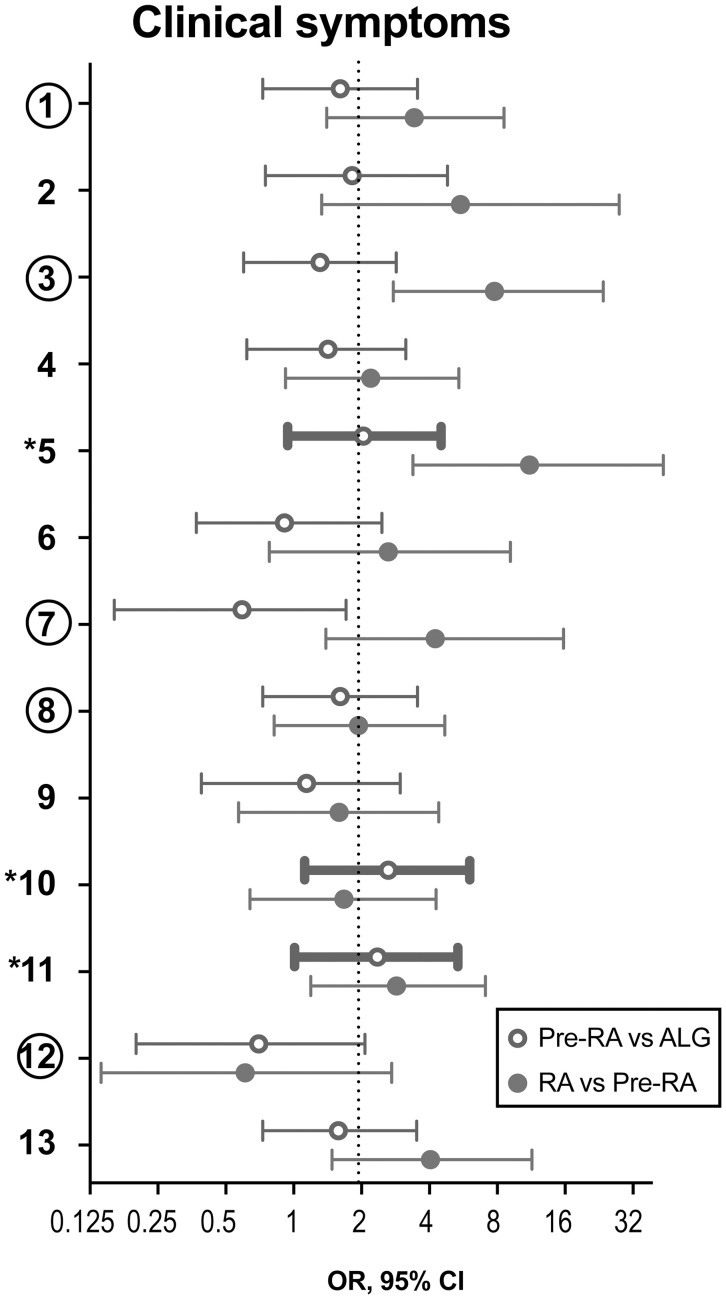
Clinical symptoms to discriminate the pre-RA patients from arthralgia and RA patients. During the prospective follow-up of 180 patients with unexplained arthralgia (ALG), 32 patients developed RA. These patients were considered pre-RA at the time of their first visit. Clinical parameters at the first visit of the pre-RA patients are compared to the remaining ALG patients (*n* = 148) and with the patients who fulfilled RA diagnosis at the first visit (*n* = 63). Data are shown as odds ratio (OR) with 95% confidence interval. Comparison between pre-RA and ALG groups is shown with open circles, and between RA and pre-RA groups, is shown with filled circles. Thick lines indicate the parameters with OR>2.0 discriminating pre-RA from ALG. The 3 symptoms marked with asterisk distinguish pre-RA cases. The 5 symptoms marked with circles comprise by consensus the positive definition of arthralgia in progression to RA. (1) Joint symptoms onset <1 year, (2) 4–10 joints with symptoms, (3) Symptoms in MCP joints, (4) Symptoms in MTP joints, (5) Symptoms in several small joint regions, (6) Symmetric symptoms and signs, (7) Duration of morning stiffness >60 min, (8) Most severe symptoms in the early morning, (9) Improvement of symptoms during the day, (10) Increasing number of joints with symptoms over time, (11) Patient experience of swelling of small hand joints, (12) Presence of a first-degree relative with RA, (13) Local tenderness involved joints at physical examination.

**Figure 2:**
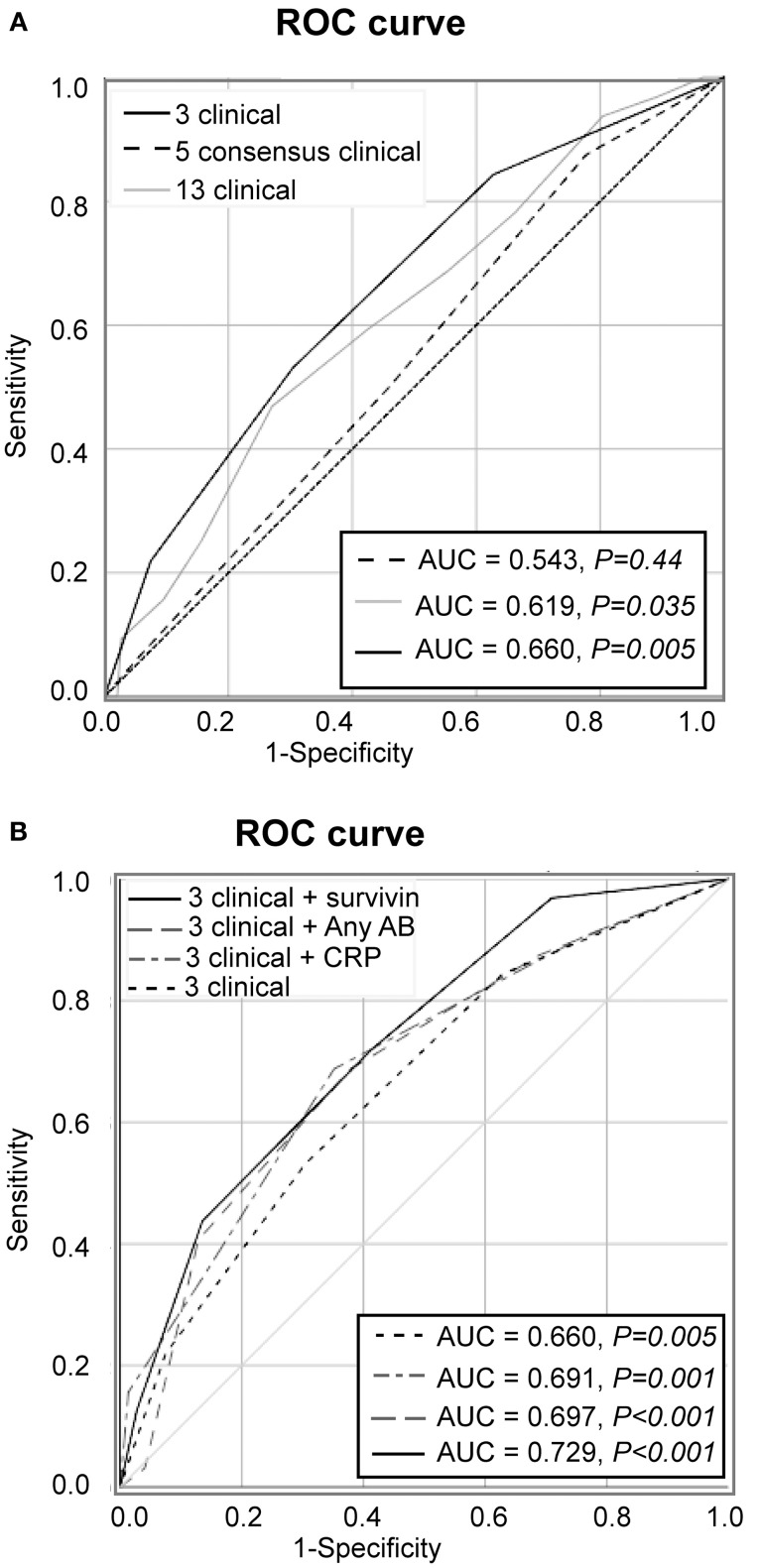
Predictive performance of clinical symptoms for transition to RA. **(A)** Receiver operator characteristics (ROC) analysis of new RA cases among the patients with arthralgia (*n* = 180) was based on 13 clinical symptoms added unweight (gray line), on the 5 symptoms comprising by consensus the definition of clinically suspect arthralgia (dashed line) or on the 3 symptoms discriminating between pre-RA and arthralgia (solid black line). **(B)** The model with 3 clinical parameters in combination with survivin (solid black line), with CRP (scattered-dash gray line), with anyAB (dash gray line) or 3 clinical parameters alone (scattered line line). Area under the ROC (AUC) of the model is shown in the Table. Diagonal line represents the reference.

During the prospective follow-up for the period of 48 months, 32 (17.8%) of 180 patients with unexplained arthralgia developed arthritis and fulfilled the EULAR/ACR 2010 criteria. To analyse if clinical symptoms at the first visit were different in these pre-RA patients and could recognize them among the arthralgia group (Figure 1), the overall frequency of those symptoms was compared. The prevalence of the symptoms appeared to be significantly higher in pre-RA patients compared to the remaining arthralgia group (*P* < 0.001), which put the pre-RA group in the intermediate position between arthralgia and RA. To assess the predictive power of the clinical parameters at the first visit for the development of RA, the ROC curve was constructed. The AUC confirmed ability of the given clinical parameters to separate pre-RA and arthralgia patients (AUC = 0.619, *P* = 0.035, Figure 2A).

Aiming to improve specificity of the model we mapped the individual symptoms within the pre-RA group and compared them to the patients with arthralgia and RA (Figure 1). This mapping revealed that the joint symptoms showed a bidirectional prediction pattern and some of the symptoms could negatively affect discriminative ability of the model in total. The next step of the analysis was focused on the positive prediction and included three joint symptoms with the odds ration above 2.0 between the pre-RA and arthralgia groups. This combined the clinical symptoms with strong association to RA and included the symptoms in several small joints (symptom 5), increasing number of joints with symptoms over time (symptom 10) and patient's experience of swelling of small hand joints (symptom 11). The symptoms are identified here as being most likely present in pre-RA patients at their first visit to rheumatologist. Using these variables unweight, we achieved an improvement of the ROC curve (AUC = 0.660, *P* = 0.005, Figure 2A). Importantly, the combination of those 3 pre-selected clinical parameters significantly improved the specificity for new RA compared to any 3 symptoms in the entire set (92.6 vs. 19.6%). The positive predictive value was 38.9%, 95% CI: 21.1-60.2.

We also studied the performance of the five clinical symptoms comprising a positive definition of arthralgia suspicious for progression to RA (Figure 2A). However, that combination of clinical symptoms provided no significant separation of the AUC for pre-RA patients from the reference line and presence of any 3 of these symptoms resulted in specificity of 83% for pre-RA, which was identical to the specificity reported by the EULAR taskforce cohort (18).

### Clinical associations of serological biomarkers

**Figure 3:**
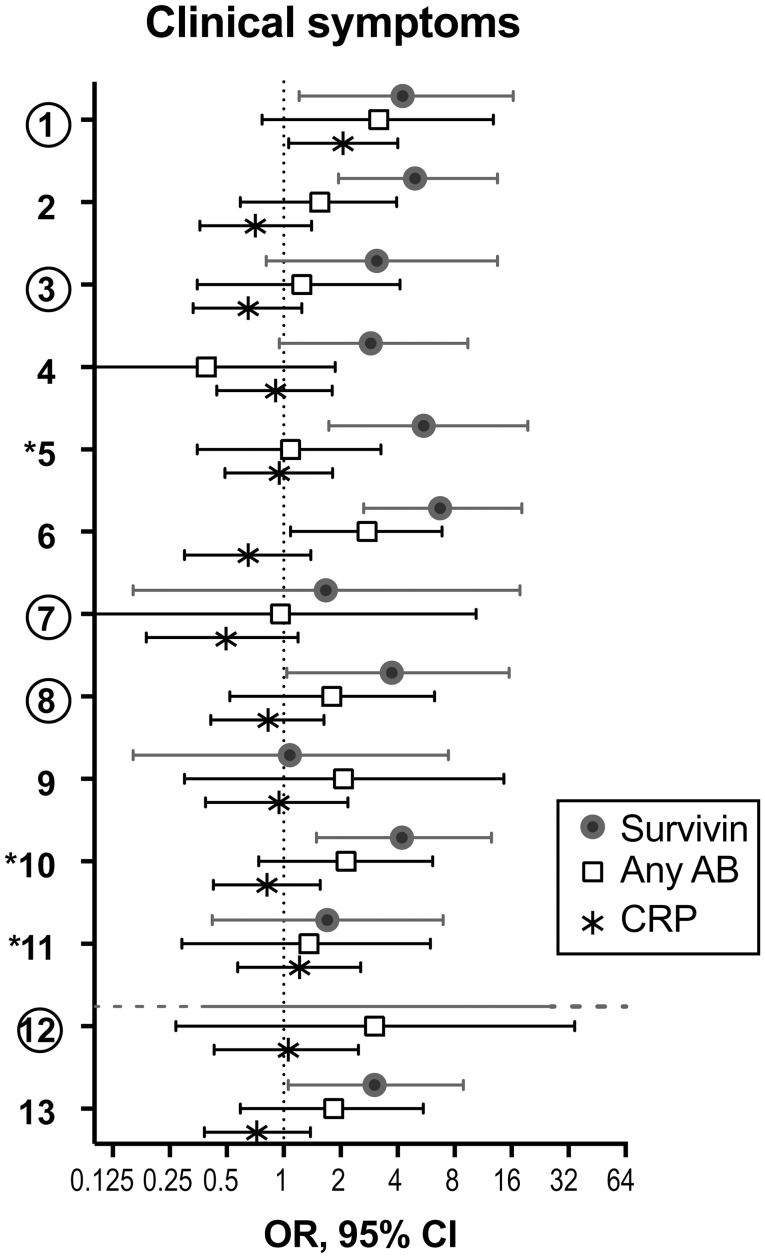
Adherence of serological biomarkers to clinical symptoms of arthralgia. To study if serological biomarkers associate with clinical symptoms of arthralgia, the prevalence of each symptom was compared among patients with and without survivin (filled circles, *n* = 61 vs. 119), any antibody (anyAB, RF and/or ACPA) (open squares, *n* = 53 vs. 127) or CRP (asterisk, *n* = 54 vs. 126). Odds ratio (OR) with 95% confidence interval (CI) is presented for each clinical symptom. The 3 symptoms marked with asterisk distinguish pre-RA cases. The 5 symptoms marked with circles comprise by consensus the positive definition of arthralgia in progression to RA. (1) Joint symptoms onset <1 year, (2) 4–10 joints with symptoms, (3) Symptoms in MCP joints, (4) Symptoms in MTP joints, (5) Symptoms in several small joint regions, (6) Symmetric symptoms and signs, (7) Duration of morning stiffness >60 min, (8) Most severe symptoms in the early morning, (9) Improvement of symptoms during the day, (10) Increasing number of joints with symptoms over time, (11) Patient experience of swelling of small hand joints, (12) Presence of a first-degree relative with RA, (13) Local tenderness involved joints at physical examination.

Since recent validation of the EULAR definition of arthralgia in progression to RA indicated a need to combine clinical symptoms with biomarkers (18), we studied which of the clinical joint symptoms discriminated for the presence of RA-specific antibodies, RF and ACPA, elevated CRP, and survivin. Comparison between the incidence of individual clinical symptoms in the arthralgia cohort separated by serological biomarkers, taken individually is presented as OR (Figure 3). We found that survivin was significantly associated with seven of the studied joint symptoms (1, Joint symptoms onset <1 year; 2, 4–10 joints with symptoms; 5, Symptoms in several small joint regions; 6, Symmetric symptoms and signs; 8, Most severe symptoms in the early morning; 10, Increasing number of joints with symptoms over time; 13, Local tenderness involved joints at physical examination). Interestingly, only two of the symptoms (5, symptoms in several small joint regions, and 10, increasing number of joints with symptoms over time) may be found among those, which included in the positive definition of arthralgia in progression to RA (17). Yet another survivin-associated symptom (1, joint symptoms onset <1 year), which is also a part of the positive definition, appeared to be associated with elevated CRP. A different survivin-associated symptom (6, Symmetric symptoms and signs) was found to be frequent in the presence of RA-specific antibodies. In total, the confidence intervals in survivin-positive pre-RA patients appeared on the right-hand side of the scale and indicated its strong association with clinical symptoms of arthralgia and pre-RA state compared to RA-specific antibodies and CRP.

### Improvement of the predictive potential of clinical algorithm

Aiming to further improve the sensitivity and predictive potential of the algorithm, the pre-selected clinical parameters discriminating between pre-RA and arthralgia (clinical symptoms 5, 10, and 11) were enriched with one new variable at a time and new models were analyzed. The variables included in the new models were elevated CRP, presence of ACPA and/or RF and presence of survivin (Figure 2B). The combination of 3 clinical parameters and serum survivin resulted in a strong improvement of the ROC curve (AUC = 0.729, *P* < 0.001; sensitivity 43.8%). Enrichment of the model with CRP or the presence of ACPA and/or RF (any AB) improved the ROC curve (Figure 2B) and provided the sensitivity of 37.5 and 40.6%, respectively.

The sensitivity and specificity levels for a combination of clinical and serological parameters in pairs are shown in Table 2. It may be appreciated that sensitivity of models improved with increasing number of parameters, which is in agreement with a diversity of parameters and symptoms within an individual arthralgia patient. The models containing survivin were associated with better predictive performance independently of other components. The model combining 3 clinical and 3 serological parameters resulted in AUC identical to the one obtained in the model combining 3 clinical parameters with survivin, any AB and female older than 50 years (AUC = 0.768, *P* < 0.001).

**Table 2:** Predictive performance of clinical joint symptoms and serological parameters.

	**ROC, AUC 95% CI**	***P-*value**	**Sens. %**	**Spec. %**	**PPV, %**
3 clinical	0.660 0.556–0.763	0.005	21.9	92.6	38.9
+ Survivin+CRP	0.756 0.665–0.847	<0.001	56.3	80.4	38.3
+ Survivin+AnyAB	0.745 0.654–0.836	<0.001	59.4	78.4	37.3
+ CRP+AnyAB	0.721 0.618–0.825	<0.001	56.3	80.4	38.3
+ Survivin+CRP+AnyAB	0.768 0.676–0.860	<0.001	68.8	70.9	33.8
+ Survivin+AnyAB+female >50y	0.768 0.674–0.860	<0.001	68.8	71.6	34.4

*Three clinical symptoms (symptoms in several small joint regions, increasing number of joints with symptoms over time, and patient experience of swelling of small hand joints) distinguished between the pre-RA (n = 32) and arthralgia (n = 148) patients by the odds ratio >2.0. To improve the sensitivity for RA, these symptoms were enriched by other parameters one-by-one and the ROC curve analysis was performed. Sensitivity and specificity of each model is calculated on the level of 3 positive parameters*.

**Figure 4:**
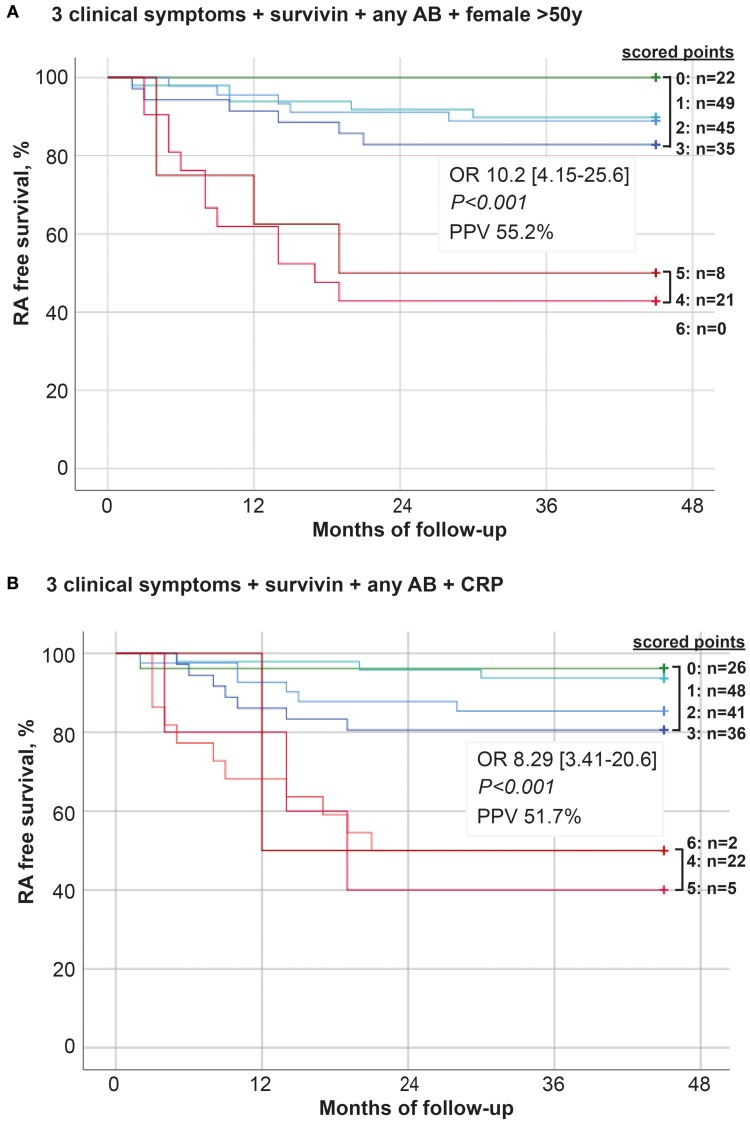
Development of new RA among arthralgia patients. One-eighty patients with unexplained arthralgia were followed for 48 months. During the follow-up time 32 of the patients developed RA. The model predicting transition from arthralgia to RA was built on 6 parameters unweight: **(A)** symptoms in several small joint regions, increasing number of joints with symptoms over time, patient experience of swelling of small hand joints, presence of survivin, presence of any antibody (AB, RF, and/or ACPA) and female >50 years. **(B)** symptoms in several small joint regions, increasing number of joints with symptoms over time, patient experience of swelling of small hand joints, presence of survivin, presence of any antibody (AB, RF, and/or ACPA) and elevated levels of CRP. The Kaplan-Meier curves were constructed and depict development of new RA cases in patients with these parameters present individually or in unrestricted combinations. OR and PPV was calculated at 48 months between patients with ≤ 3 and ≥4 parameters.

The Kaplan-Meyer curves were built to depict development of new RA cases among the arthralgia patients with the presence of any of the parameters. The algorithm comprised 3 clinical symptoms (5, symptoms in several small joint regions, 10, increasing number of joints with symptoms over time, or 11, patients experience of swollen small hand joints), the presence of survivin, ACPA and/or RF (any AB) and female older than 50 years. In the alternative algorithm, the last parameter was substituted with CRP. The parameters could be present individually or in combinations (Figures 4A,B). The evaluation demonstrates higher probability for RA development in patients combining several of the parameters. If present in combination of more than 3, theses parameters significantly increased the probability and shortened time to RA. This selection strategy permitted PPV of 55.2%, 95% CI: 36.0–73.0 (A). The alternative algorithm permitted PPV of 51.7%, 95% CI: 33.9-70.1.

## Discussion

Patients with joint complain which could be neither explained by joint swelling nor by other known disease, is frequently a subject for rheumatologic assessment. In this prospective study, patients with unexplained arthralgia comprised 10.3% of all the first-visit rheumatology patients in Gothenburg. The prospective follow-up of those arthralgia patients revealed that as much as every fifth patient developed joint swelling and could be classified as RA within coming 48 months. Rheumatology service in Gothenburg is structurally organized around the academic practice of the University Hospital. Any general practitioner or other specialist may send a referral directly to the rheumatology clinic. Additionally, any patient with musculoskeletal complains will meet a rheumatologist within 3 months after own referral. A limited number of private rheumatology practices in Gothenburg make us to believe that the first-visit cohort described in this study is representative to identify most of the individuals in Gothenburg with recent onset arthritis and those with persistent arthralgia. This belief is supported by the prevalence of RA diagnosis at the first visit, which is positioned within the expected prevalence of 0.1–0.5% (54). Consequently, tight follow-up of the arthralgia patients enlarged the annual RA cohort by additional 51%.

This single center cohort was collected through the first-visit records of the routine rheumatologic examination and showed that the clinical symptoms for RA and UA patients differ significantly from those in patients with arthralgia. We were surprised to learn that three definite symptoms of the entire set distinguished pre-RA cases at the stage of arthralgia with specificity of 92.6% and had PPV of 38.9%. In our study, the collection of clinical symptoms was done in a careful evaluation of medical records by independent investigators, who had no knowledge of clinical outcome in individual patients. Additionally, the records reflected a routine rheumatologic visit outside the pre-defined setting of clinical trial. This way of collecting clinical information reduces the bias introduced by the examining rheumatologist. It gives neither a chance to clarify a goal of evaluation nor to lead the answer to a specific location of symptoms. Taken together, the evaluation strategy of our study strengthens reliability of the reported outcomes.

In the final algorithm, the probability rate for RA development depended on the serological prolife of the patients. Importantly, we observed that the presence of high survivin levels in serum had distinct association with most of the clinical joint symptoms listed by the rheumatology expert taskforce (17), while no such an association was found for the autoantibodies or CRP. Evaluation of clinical symptoms in relation to serological parameters presented a diverse picture, in which survivin was identified adherent to most of them and served a common link to the symptoms associated with both CRP and autoantibodies. This provides further support to the hypothesis that survivin is critical for biological processes foregoing onset of RA (40, 50, 52). Translating those experimental findings into clinical practice, the adherence of early joint symptoms to survivin shows an independent added value of survivin measurement to identify arthralgia patients, that eventually develop RA. It identifies the patients, which if found early, may favor of targeting interventions to prevent imminent RA. Recent results obtained in material of the SWEFOT trail provide evidence that sero-conversion of survivin is achievable during treatment and that it is favorable for clinical outcome of patients with early RA (47). Today, RA-specific antibodies are used as an ultimate indication of arthralgia patients at risk to develop RA and are justified for interventional treatment. However, none of the two treatment trials comprising autoantibody-positive arthralgia patients reached the primary endpoint of preventing arthritis (10, 56). In sharp contrast to survivin, the reduction of autoantibodies during treatment was not translated into clinical outcome.

The best predictive performance was reached by using a combination of pre-selected clinical parameters with female gender, survivin and autoantibodies. Present in combination of more than 3 symptoms, they aided sensitive and specific enough recognition of pre-RA patients at the stage of arthralgia. These findings confirm the observations done in a different longitudinal study of arthralgia patients, which has shown that the patients with less than 3 clinical items have a low risk of progression to RA (18). The measures of C-reactive protein in our arthralgia cohort had the sensitivity, which was inferior to survivin and the autoantibodies. CRP had poor adherence to clinical symptoms of arthralgia. Unexpectedly, CRP was a valuable complement in the final predictive algorithm. On the one side, the presence of increased acute-phase proteins is included in the classification criteria for RA and formally facilitates the diagnosis. On the other side, the presence of acute-phase proteins in arthralgia may indicate dawning of RA and will be naturally confirmed by more distinct clinical symptoms (57).

Due to its design, the study reflects certain limitations in the follow-up strategies of patients with arthritis in the real-life setting. One of which appeared to be the inability to identify the exact time point for conversion from UA to RA. This occurred due to the traditions of active anti-inflammatory treatment in rheumatology, where initiation of treatment with disease modifying anti-rheumatic drugs and corticosteroids occurred at least in a part of UA patients. This latter action, however, improves health outcomes.

Taken together, this study shows that a distinct combination of clinical parameters including symptoms in several small joint regions, increasing number of joints with symptoms over time, and patient's experience of swelling of small hand joints may with high sensitivity distinguish the arthralgia patients at preclinical stage of RA. Grouping those clinical parameters with information about serum levels of survivin, female gender or autoantibodies improves the sensitivity to prospective transition from arthralgia to RA and increases diagnostic precision favorable for early healthcare strategies.

## Declarations

### Ethics approval

The study protocol was reviewed and approved by the Ethical Review Boards of Gothenburg. All methods used in this study were carried out in accordance with relevant Swedish guidelines and regulations and following the Good Clinical Practice. The study is based on the routine rheumatologic evaluation and the results of blood analysis after the referral from general practitioners, which does not require the informed consent. The informed consent was obtained from all subjects in case of additional clinical visits or blood sampling.

## Availability for data and materials

The datasets used and/or analyzed during the current study available from the corresponding author on reasonable request.

## Author contributions

MB conceiving the study. MB and RP collecting patient material. ME and MT laboratory analyses. ME, MT, and RP statistical analyses. ME, MT, and MB writing the paper. All authors have read and approved submission of the manuscript in its present form.

### Conflict of interest statement

The authors declare that the research was conducted in the absence of any commercial or financial relationships that could be construed as a potential conflict of interest.
